# Quantitative Characterization of Non-Classic Polarization of Cations on Clay Aggregate Stability

**DOI:** 10.1371/journal.pone.0122460

**Published:** 2015-04-15

**Authors:** Feinan Hu, Hang Li, Xinmin Liu, Song Li, Wuquan Ding, Chenyang Xu, Yue Li, Longhui Zhu

**Affiliations:** Chongqing Key Laboratory of Soil Multi-Scale Interfacial Process, College of Resources and Environment, Southwest University, Chongqing, China; Instituto de Tecnologica Química e Biológica, UNL, PORTUGAL

## Abstract

Soil particle interactions are strongly influenced by the concentration, valence and ion species and the pH of the bulk solution, which will also affect aggregate stability and particle transport. In this study, we investigated clay aggregate stability in the presence of different alkali ions (Li^+^, Na^+^, K^+^, and Cs^+^) at concentrations from10^−5^ to 10^−1^ mol L^−1^. Strong specific ion effects on clay aggregate stability were observed, and showed the order Cs^+^>K^+^>Na^+^>Li^+^. We found that it was not the effects of ion size, hydration, and dispersion forces in the cation–surface interactions but strong non-classic polarization of adsorbed cations that resulted in these specific effects. In this study, the non-classic dipole moments of each cation species resulting from the non-classic polarization were estimated. By comparing non-classic dipole moments with classic values, the observed dipole moments of adsorbed cations were up to 10^4^ times larger than the classic values for the same cation. The observed non-classic dipole moments sharply increased with decreasing electrolyte concentration. We conclude that strong non-classic polarization could significantly suppress the thickness of the diffuse layer, thereby weakening the electric field near the clay surface and resulting in improved clay aggregate stability. Even though we only demonstrated specific ion effects on aggregate stability with several alkali ions, our results indicate that these effects could be universally important in soil aggregate stability.

## Introduction

Aggregate stability is important because it not only affects many properties and processes of soil, but is also related to environmental problems. Soil aggregates are mainly formed by flocculation, cementation, and rearrangement of soil particles, including clay colloids, organic matter, and oxides [1,2], in which various factors and processes are involved. Understanding soil aggregate stability is crucial because it is related to many properties and processes, for example, infiltration capacity, hydraulic conductivity, solute transport, the carbon cycle, tilth, erodibility, soil degradation, and environmental pollution [1, 3–10].

Aggregate stability is affected by many factors. In particular, the interaction between the solid phase and the liquid phase in the soil system can greatly affect the stability of soil aggregates [11,12]. Solid soil materials, such as clays, organic matter, and Fe and Al oxides, have important effects on soil aggregate stability, and these factors have been investigated by many researchers [2,4,8]. The liquid soil phase, which mainly contains water, and the chemical composition can also greatly affect aggregate stability. Aggregate breakdown caused by changing the soil water content and chemical composition may derive from a variety of physical and physical–chemical mechanisms [10,11,13]. In these cases, the interaction forces between soil colloidal particles are always described, and they are considered to explain the processes of soil particle aggregation and aggregate breakdown [11,14]. It has been confirmed that the strong electric repulsive force (∼1000 atm) resulting from the particle surface plays an important role in soil colloid interactions [15,16]. Some studies have reported that increasing the electrolyte concentration of the bulk solution and/or adding high-valence ions to the bulk solution can decrease the electric field and thus improve the stability of aggregates [16,17]. These conclusions can be well understood by Derjaguin–Landau–Verwey–Overbeek (DLVO) theory. Based on this theory, ions with the same valence and concentration will have the same ability to maintain the aggregate stability. Nonetheless, from experience, we know that aggregate stability is influenced by not only the ion concentration and valence but also by the ion species. Levy and Torrento [18] found that K^+^ could limit clay dispersion and maintain macroaggregate stability, while Na^+^ could accelerate aggregate breakdown. Levy and Van Der Watt [19] observed that ions had different abilities to maintain the aggregate stability, and followed the sequence Ca^2+^>K^+^>Na^+^. To this phenomenon, Amézketa [17] remarked in his review paper that there seemed to be relationships between aggregate stability and the Hofmeister series that indicated the decreasing order of cations in promoting flocculation is Ca^2+^>Mg^2+^>K^+^>Na^+^. However, what happened next about Hofmeister effects on aggregates stability does pay little attention to soil science hitherto.

Specific ion, or Hofmeister, effects are universal in physics, colloid and surface science, biology, and geology. The Hofmeister phenomenon was firstly reported in the 1880s, where Hofmeister and coworkers observed that different ions processed different abilities to disnature hen egg proteins. Based on this, ions were ordered by the concentration required to precipitate proteins. This type of sequence is called a Hofmeister series. In general, Hofmeister series are not only concerned with proteins, but are also believed to apply to a wide range of phenomena [20]. For example, they are observedin protein precipitates, membrane transport across membranes, pH measurements, ion exchange equilibrium experiments, zeta potentials, surface tension, and colloid stability [21–27]. At first, it was thought that specific ion effects were not important and even could be ignored. However, especially in the last 10 years, specific ion effects in physical chemistry have attracted extensive interest from scientists in different fields, and have been recognized as important in Mendel’s work on genetics [28]. For a long time, physical science would lose its certainty in attempting to explain some biological phenomenon [28]. Specific ion effects are fundamentally important and may remove the barriers between the physical and biological sciences. Despite its fundamental importance in many fields, the molecular-level understanding of the mechanism behind this effect is still the subject of intense debate.

Many theories have been proposed to elucidate the origins of specific ion effects. For a long time, the ion hydration hypothesis was widely accepted to clarify the mechanism of specific ion effects [29,30]. In addition, hydration effects were thought to be the main reason for the different ability of ion exchange in soil science. However, these effects have been disproved by recent advances in experimental and theoretical studies on specific ion effects [29,31–34]. Although many studies have emphasized the important role of quantum fluctuation (dispersion) forces in specific ion effects [35,36], these forces only become important at high electrolyte concentrations (>0.1 mol L^−1^) when the electrostatic force is completely screened [36–41]. This means that specific ion effects will not occur at low electrolyte concentrations. However, a few studies have recently shown that specific ion effects can also be found at low electrolyte concentrations and are amplified with decreasing electrolyte concentration [25,38,42–44]. Recently, in our group, specific cation effects at low electrolyte concentration were investigated by systematically changing cation species in cation-exchange equilibrium experiments [25,42]. We demonstrated that with decreasing cation concentration, the differences in the selectivity coefficients among different ions were amplified and specific ion effects were obvious at low electrolyte concentrations. In addition, we found that the strong polarization effect resulting from the coupling between the huge electric field (>10^8^ Vm^−1^) of the clay surface and ionic quantum fluctuation played a crucial role in the cation-surface interaction. Tian et al. [44] investigated specific ion effects on colloidal particle aggregation, and pointed out that compared with classic induction theory, only the strong polarization effect can give a rational explanation of the observed specific ion effects. A similar conclusion was also reached by Noah-Vanhoucke and Geissler [45]. Note that, nanoscale surfaces and colloidal particles (e.g., DNA, proteins, cells, bacteria, metal oxides,and clay) in solution can establish a strong electric field (usually >10^8^ V m^−1^) that spreads out from the surface to several nanometers from the surface [25]. The strong electric field can change the electron cloud configuration of surrounding ions of the charged surface, and thus result in much stronger polarizability of ions than classic polarization effects, and it was referred to as non-classic polarization [46]. Xu et al. [46] found that, the non-classic polarization would strongly influence clay aggregate stability, but they did not quantitatively characterize those effects.

Aggregate stability is a hot topic because it is not only of interest to agricultural scientists but also to environmental experts. Microparticles released from soil aggregate breakdown or because of decreasing aggregate stability and mineral dissolution can cause serious environmental problems [42,47,48]. Specific ion effects on mineral dissolution have been investigated by many groups [49–52]. However, to the best of our knowledge, similar studies on aggregate stability are rare. Therefore, the objective of this study was to identify specific ion effects on aggregates stability, explore and quantitatively characterize the strong non-classic polarization effects on aggregates stability.

## Materials and Methods

### Materials

Purified montmorillonite was supplied by WuHuaTianBao Mineral Resources Co., Ltd. (Inner Mongolia, China), and was used as the experimental material. X-ray diffraction showed that the purified mineral was pure montmorillonite. The surface properties were evaluated by a combination of methods [53]. The specific surface area was 716 m^2^g^−1^ and the surface charge number of the montmorillonite was 84.8 cmol_c_kg^−1^.

We used Li^+^ (LiNO_3_), Na^+^ (NaNO_3_), K^+^ (KNO_3_), and Cs^+^ (CsNO_3_) to characterize specific ion effects on clay aggregate stability. Herein, it should be noted that using Li^+^, Na^+^, K^+^, and Cs^+^ in this study was not because soil contains or only contains these cation species, but because they are the best selection to characterize the existing specific ion effects in soil [30,49,54]. The LiNO_3_, NaNO_3_, KNO_3_, and C_S_NO_3_ salts used were of analytical grade and used as received. All solutions were prepared at room temperature using fresh deionized water with a resistivity of 18.25 MΩ cm. The concentrations of the salts varied from 10^−5^ to 10^−1^mol L^−1^. To study cation-specific phenomena, we used nitrate salts of all the cations to avoid the influence of different anions. Nitrate makes strong acids, therefore the solutions of the corresponding salts have nearly neutral pH values. Thus, pH adjustment in this study was not required.

### Sample Preparation

To quantitatively investigate specific ion effects on aggregate stability, and the role of the strong electric field resulting from clay particle surfaces, it is important to distinguish which ions in clay are responsible. Therefore, the raw montmorillonite was saturated with the given ion species to replace the originally adsorbed impurity ions. In this way, the characteristics of the obtained aggregates could be analyzed under identical conditions for the purpose of direct comparison.

The X^+^-saturated samples (X = Li, Na, K, or Cs) were obtained following the procedure described by Li et al. [16]. Here, we take Na^+^-saturated aggregates as an example. Eight hundred grams of purified montmorillonite was weighed into a 5-L beaker containing 4L of 0.5 mol L^−1^ NaNO_3_. First, the suspension was successively washed by dispersion, agitation (24 h), centrifugation, and decantation with three portions of 4 L 0.5 mol L^−1^ NaNO_3_, and then washed with deionized water to remove excess salts. Each cation-saturated soil sample was then dried at a temperature of 333K, crushed, and passed through a 5-mm sieve to collect 1- to 5-mm diameter aggregates for the experiments. All of the saturated clay aggregates were prepared using the similar procedure.

### Aggregate stability experiments

In natural events, the soil electric field influenced by the electrolyte concentration in the bulk solution is mainly controlled by the wetting–drying cycle. Here, we directly changed the electrolyte concentration to adjust the soil electric field strength. In our experiments, the concentrations for each system were set to 10^−5^, 10^−4^, 10^−3^, 10^−2^, and 10^−1^ mol L^−1^.

Aggregate stability can be determined by measuring the amount of small particles, including microparticles (<10, <5, and <2 μm) released from the macroaggregates (1–5 mm). To investigate specific ion effects based on the mechanism of the physical chemistry interaction, combined static sedimentation methods and the pipette method were used to measure the amount of small particles. We believe that this method is more appropriate than the wetting sieves method to reduce the external disturbance forces.

For the aggregate stability experiments, we will again take Na^+^-saturated aggregates as an example. A specific mass of Na^+^-saturated clay aggregates (20 g) was weighed into cylinders (500 mL) containing 10^−5^, 10^−4^, 10^−3^, 10^−2^, and 10^−1^ mol L^−1^ NaNO_3_ solutions. The solution temperature was 298 K. After aggregate breakdown by internal forces, the released particles were evenly distributed in the cylinders by carefully turning the cylinders up and down. Then, the mass percentage of the released small particles with diameters <*d* (*d* = 10, 5, and 2 μm) with respect to the total mass of aggregates could be measured via the pipette method. The same procedure was used for the other experiments, except that the solutions in the cylinders were LiNO_3_, KNO_3_, and CsNO_3_.

## Results and Discussion

### Effect of ion concentration and strong electric field on the stability of clay aggregates

**Fig 1:**
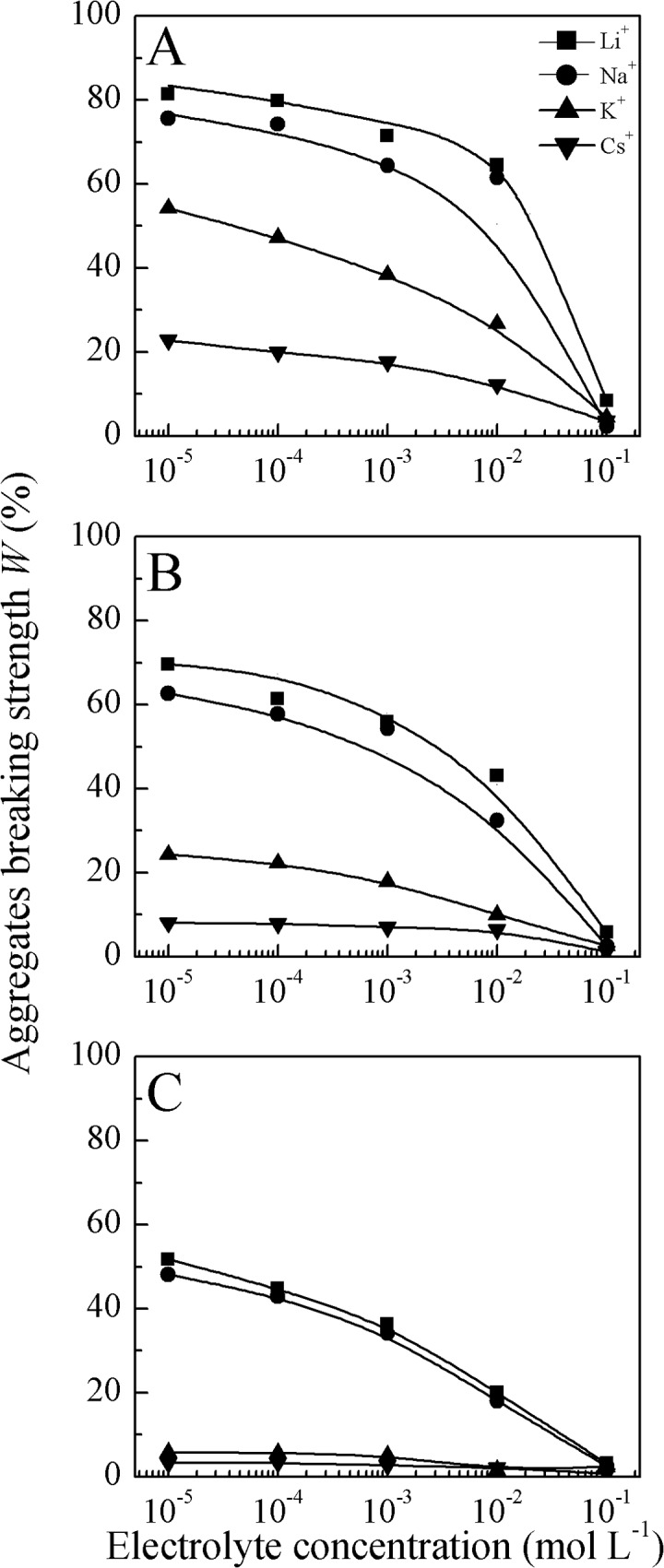
Relationship between aggregates breaking strength and electrolyte concentrations (A, B and C represent released small particles of *d*<10, 5 and 2 μm, respectively).

Fig 1 shows the aggregate breaking strength (*W*) dependence on electrolyte concentration for LiNO_3_, NaNO_3_, KNO_3_, and CsNO_3_. The most striking observation was the change in the aggregate breaking strength with electrolyte concentration. After aggregate breakdown, the percentages of small particles (*d*<10, 5, and 2 μm) released from macroaggregates (1–5 mm) are shown in Fig 1. In all cases, the aggregate breaking strength increased with decreasing electrolyte concentration from 0.1 to 10^−5^ mol L^−1^. For example, at a high electrolyte concentration of 0.1 mol L^−1^, there were almost no small particles (including microaggregates) released in all cases, indicating that clay aggregates are quite stable and no aggregate breakdown occurred. In contrast, at a low electrolyte concentration of 10^−4^ mol L^−1^, the aggregate breaking strengths for *d*<5 μm were 61.36%, 57.77%, 22.21%, and 7.86% for the LiNO_3_, NaNO_3_, KNO_3_, and CsNO_3_ systems, respectively. It is clear that the clay aggregate stability was dependent on the electrolyte concentration.

In all cases in the present study, with decreasing electrolyte concentration, the aggregate stability decreased. This is in agreement with commonly observed results [11,16,55]. Actually, in the typical process of aggregate breakdown, a decrease in electrolyte concentration or ionic strength of the aqueous solution leads to system destabilization, and thus decreases aggregate stability. This is essentially because of an increase in the inter-particle electrostatic repulsive forces, which is responsible for the decrease in aggregate stability [56–59], which can be explained by the well-known DLVO theory. In classic DLVO theory, the electric field of particles with like charges generates a repulsive force to resist soil particle aggregation, and this force can be strengthened by decreasing the electrolyte concentration. That is, decreasing the electrolyte concentration will increase the surface potential (in absolute value) and amplify the electric field, resulting in aggregate breakdown [16].

According to the double layer theory, the electric field strength at position *x* in the diffuse layer in Z:Z type of electrolyte solutions could be expressed as:

$$E\left(x\right)=\sqrt{\frac{8RTc_{0}}{\varepsilon_{0}\varepsilon_{r}}}\sinh\frac{ZF\phi \left(x\right)}{2RT}=\frac{\sigma \left(x\right)}{\varepsilon_{0}\varepsilon_{r}}$$ 1

Where *E*(*x*) (V m^-1^) is the electric field strength around soil particles; *x* (m) is the distance from particles surface, *R* (J mol^-1^ K^-1^) is the universal gas constant; *T* (K) is the absolute temperature; *c*_*0*_ (mol m^-3^) is the equilibrium concentration of the 1:1 type electrolyte in bulk solution; ε_*0*_ is the permittivity of free space which equals 8.85× 10^–2^ C^2^ J^-1^ m^-1^; and ε_*r*_ is the static relative permittivity of water; *Z* is the valence of counterion; *F* (C mol^-1^) is the Faraday constant; σ(*x*) is the charge density at the position of *x*, which can be calculated by Eq (2); *φ*(*x*) is the potential at the position of *x* in diffuse layer and can be calculated by Eq (3).

$$\sigma \left(x\right)=\sqrt{8RT\varepsilon_{0}\varepsilon_{r}c_{0}}\sinh\frac{ZF\phi \left(x\right)}{2RT}$$ 2

$$\phi \left(x\right)=\frac{4RT}{F}\tanh^{-1}\left(ae^{-\kappa x}\right)$$ 3

where

$$a=\tanh\left(\frac{ZF\phi_{0}}{4RT}\right)\;\text{and}\;\kappa =\sqrt{\frac{2Z^{2}F^{2}c_{0}}{\varepsilon_{0}\varepsilon_{r}RT}}$$

where a is temporary parameter; *φ*_0_ is the potential at the *x* = 0 in diffuse layer.

Therefore, for permanently charged surface, because *σ*(*x* = 0) is independent of *c*_0_, the *E*(*x* = 0) is independent of electrolyte as well. However, because *σ*(*x*) at *x*>0 is strongly dependent of *c*_0_, thus *E*(*x*) is strongly dependent of *c*_0_.

**Fig 2:**
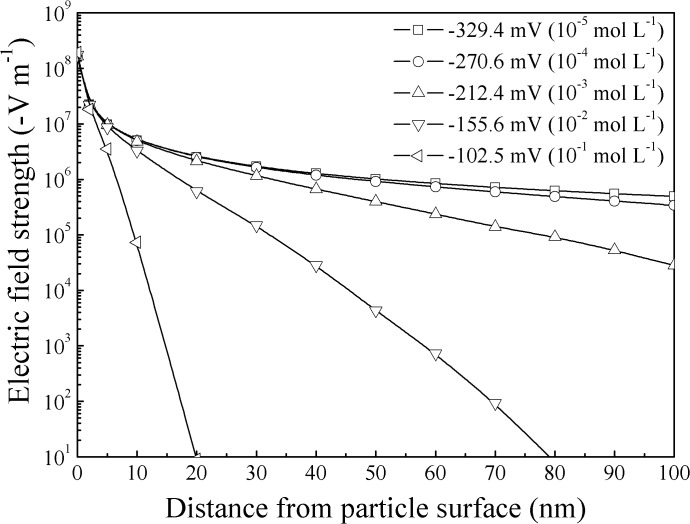
Distribution of the electric field strength from clay particle surface.

If *σ*(*x* = 0) is known, the *φ*(*x* = 0) = *φ*_0_ could be calculated from Eq (2) under given *c*_0_ conditions, and then the *φ*(*x*), *σ*(*x*) and *E*(*x*) could be calculated. Those simple calculations will surely show the *E*(*x*) at *x*>0 is the functions of both *c*_0_ and *x*. For 1:1 type of electrolyte, the calculated *E*(*x*) for the adopted material in our study is shown in Fig 2.

As shown in Fig 2, the strength of the soil electric field near the particle surface reached as high as 10^8^ V m^−1^, and, with the increase of the absolute value of the surface potential (or the decrease of electrolyte concentration), the electric field strength will increase.

The electrostatic force per unit volume between two adjacent particles could be expressed as:

$$F_{electric}=\rho \left(x\right)E\left(x\right)=\rho \left(x\right)\frac{d\phi \left(x\right)}{dx}$$ 4

where *ρ*(*x*) is the charge density at position of *x*.

Therefore the strong electric field *E*(*x*) in diffuse layer could produce strong electrostatic repulsive pressure between two adjacent particles, the corresponding electrostatic repulsive pressure could be expressed as:

$$dp_{electric}=-\rho \left(x\right)d\phi \left(x\right)$$ 5

where *p*_*electric*_ is electrostatic repulsive pressure.

Classically, the integration of Eq (5) for 1:1 type of electrolyte gives:

$$p_{electric}=2RTc_{0}\left[\cosh\left(\frac{F\phi_{d}}{RT}\right)-1\right]=RT\left[\sum_{i}c_{i\left(d\right)}-c_{i\left(0\right)}\right]$$ 6

where *φ*_(d)_ is the potential at the midpoint of two adjacent soil particles; *c*_*i*(*d*)_ is the electrolyte concentration at the midpoint of two adjacent soil particles; *c*_*i*(0)_ is the electrolyte concentration at the bulk solution.

Therefore, Eq (5) has clearly shown that, the *p*_*electric*_ is essentially the pressure arising from electric repulsive force but not entropy, although the *p*_*electric*_ could be expressed as Eq (6).

**Fig 3:**
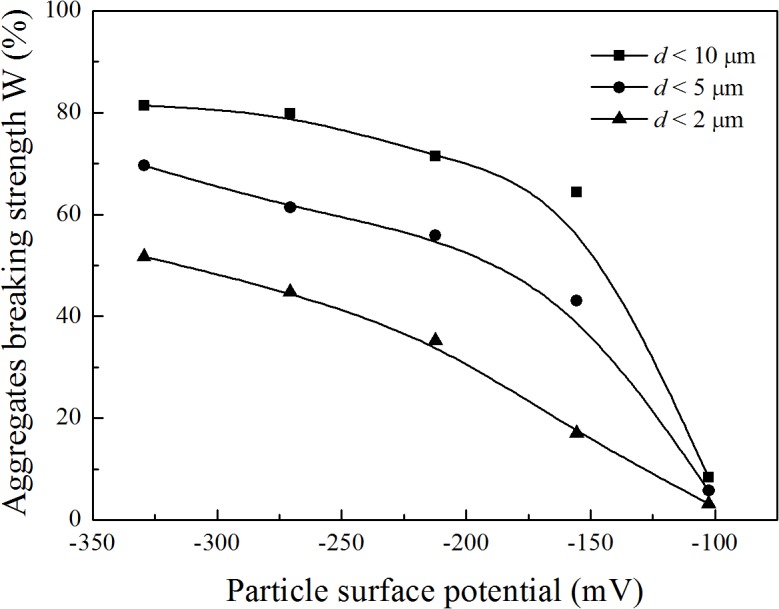
Relationship between aggregates breaking strength and particle surface potential in Li^+^ system

Fig 3 shows the relationship between aggregate breaking strength and particle surface potential in the Li^+^ system. From this figure, taking *d*<5 μm as an example, the surface potential was −270.6 mV and the aggregate breaking strength was as high as 61.36%, whereas the surface potential was −102.5 mV and the aggregate breaking strength was only 5.79%. This clearly shows that aggregate stability decreases with increasing strong electric field of the clay surface.

However, according to classic DLVO and double-layer theories, soil particles in LiNO_3_, NaNO_3_, KNO_3_, and CsNO_3_ solutions have identical electric fields, and therefore the soil aggregate stabilities should be the same, or at least resemble each other, at an arbitrary electrolyte concentration. Obviously, the differences between the aggregate stabilities among the electrolytes were significant in our experiments. Certainly, the cation diameter and its hydration diameter are different, and thus the electric field of the soil particles will be different. However, we also know that the ion diameter or hydration diameter can only produce a small second-order contribution to the electric field around the particle surface, and this contribution will disappear at low electrolyte concentrations. Fig 1 showed that, even at an electrolyte concentration of 10^−5^ mol L^−1^, there were still differences in the aggregate stabilities of the different electrolytes, which indicates that there were differences in the electric fields. Therefore, we conclude that in addition to the classic Coulomb force there must be other unknown cation–surface interactions that influence soil aggregate stability. These unknown interactions are generally referred to as specific ion effects.

### Specific ion effects on clay aggregate stability and their usual explanations

It is apparent from Fig 1 that the aggregate breaking strength in the Li^+^, Na^+^, K^+^, and Cs^+^ systems were significantly different, and changed depending on the electrolyte concentration. Specifically, when the aggregate breaking strength was measured at any given concentration, they could be ranked as Cs^+^>K^+^>Na^+^> Li^+^ when ordered by aggregate stability. This sequence is consistent with K^+^ having a greater ability to maintain aggregate stability than Na^+^ [18,19]. Therefore, the clay aggregate stability exhibits strong specific ion effects. In the present study, specific ion effects on aggregate stability were investigated for all cases in a wide range of ion concentrations from10^−5^ to 0.1 mol L^−1^. It is interesting to note that our results revealed concentration dependence of specific ion effects. Namely, when the electrolyte concentrations decreased, the aggregate breaking strengths showed noticeable differences in the four salt systems, and specific ion effects started to occur. At high electrolyte concentration (∼0.1 mol L^−1^), the aggregate breaking strengths were almost the same and did not show ion specificity for the LiNO_3_, NaNO_3_, KNO_3_, and CsNO_3_ solutions. At low electrolyte concentration (<0.1 mol L^−1^), however, there were significant differences in the aggregate breaking strengths among the LiNO_3_, NaNO_3_, KNO_3_, and CsNO_3_-saturated aggregates (Fig 1), even though the valences and salt concentrations were the same. Obviously, DLVO theory is conditionally correct in clarifying soil aggregate stability. This inconsistency is actually derived from specific ion effects, and the background mechanism of this phenomenon will be discussed in detail later.

**Fig 4:**
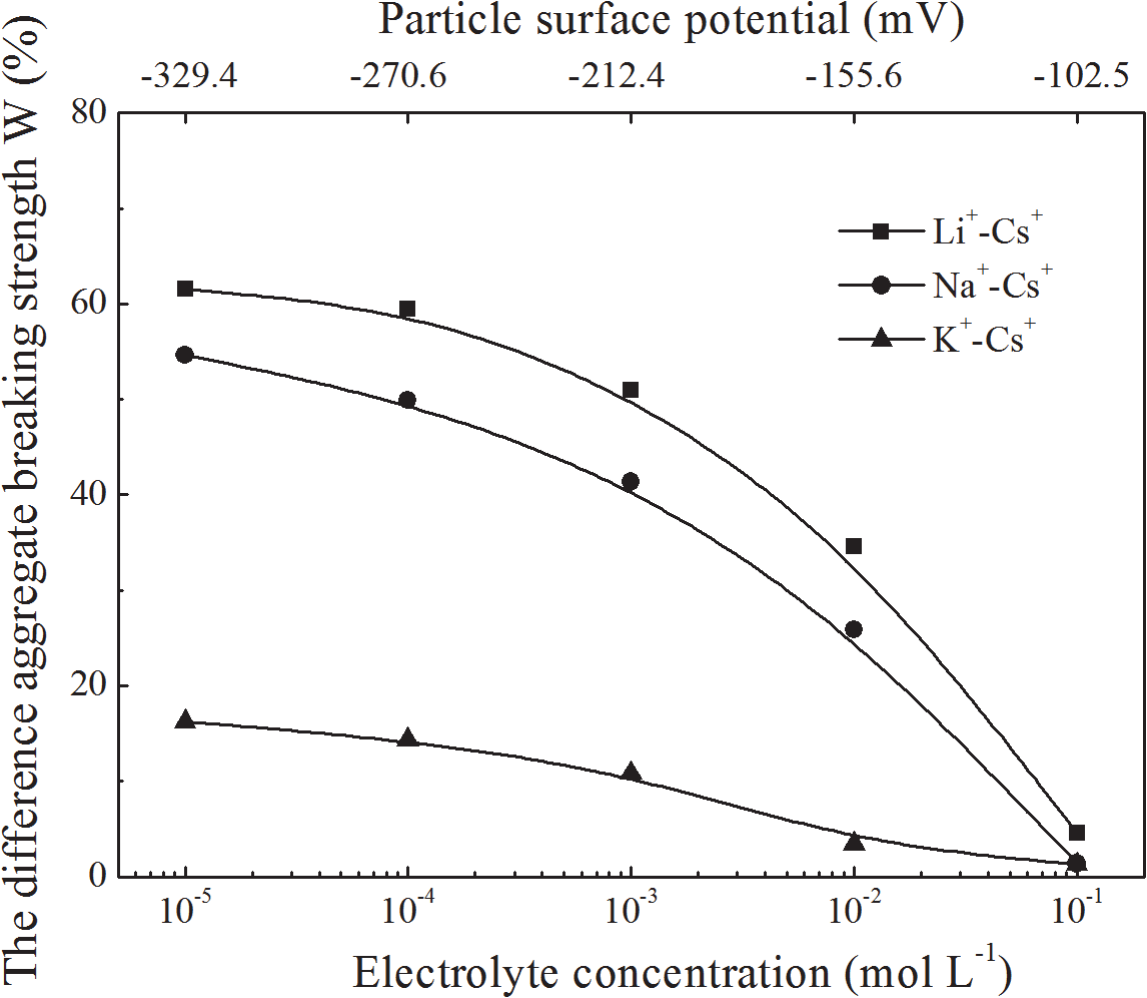
The difference of aggregates breaking strength between each two system as a function of electrolyte concentrations and surface potential.

To further illustrate specific ion effects on clay aggregate stability, the differences in the aggregate stabilities (taking the *d*<5 μm experimental data as an example) between each two electrolyte systems as a function of electrolyte concentration are shown in Fig 4. From this figure, the main characteristic of specific ion effects on clay aggregate stability was determined: the difference of aggregate stability between each two alkali cation species was related to the electrolyte concentration. Specifically, the lower the electrolyte concentration, the greater the difference in aggregate stability between two systems. For example, at a low electrolyte concentration of 10^−4^ mol L^−1^, the breaking strength of Cs-saturated clay aggregates were 59.5%, 49.9%, and 14.4% less than Li-, Na-, and K-saturated clay aggregates, respectively. However, at a high electrolyte concentration of 0.1 mol L^−1^, the corresponding differences between the aggregate breaking strengths were only 4.6%, 1.4%, and 1.3%. These results were contrary to most other findings, where specific ion effects could only be found at high electrolyte concentrations [29,31,32]. Even though the electrolyte concentrations were the same for these alkali ion solutions, the aggregate breaking strengths increased in the order Li^+^>Na^+^>K^+^>Cs^+^. As previously mentioned, a low electrolyte concentration corresponds to a high surface potential, and thus the strong electric field resulting from the clay particles surface could lead to aggregate breakdown. Clearly, specific ion effects on aggregate stability are related to changes in the surface electric field.

Although the classic diffuse double-layer and DLVO theories are still the core of colloidal and interface science, exceptions to these classic theories have been found in a wide range of phenomena where specific ion effects occurred [36,39]. Investigating the mechanism of specific ion effects is difficult, but also interesting.

It is well known that ion size and ion hydration effects are responsible for specific ion effects. Hofmeister was inclined to use the ion hydration hypothesis to interpret the origin of specific ion effects on protein stability [29,60]. It seems that proteins can be stabilized by weakly hydrated cations and destabilized by strongly hydrated cations [60]. Based on these observations, it is believed that for cation species with the same charge number, smaller ion radius must result in larger hydration radius, and thus stronger “water abstraction power”. However, hydrated ions have lower charge density than unhydrated ions. Therefore, the larger the hydrated cation radius, the lower the charge density, and thus the ability of such cations to screen the electrostatic field resulting from clay surfaces is weak. Based on this assumption, we can predict that the aggregate breaking strength follows the order Li^+^>Na^+^>K^+^>Cs^+^ when ordered by ion size and ion hydration effects. This result has been known for a long time. Parsons et al. [40] also calculated the pressure between silica surfaces in Li^+^, Na^+^, and K^+^ systems with an electrolyte concentration of 0.5 mol L^−1^, and found that the repulsive pressure between colloid particles decreased in the order Li^+^>Na^+^>K^+^. This result also agrees with recent atomic force microscope measurements between two silica surfaces [61], where it was proposed that hydration effects play an important role, illustrating the Hofmeister series. Thus, the results of experimental observations and theoretical calculations have both confirmed the importance of ion size and ion hydration effects in specific ion effects.

Ion size and ion hydration effects are reasonable to clarify the specific ion effects on aggregate stability shown in Fig 1, which showed the same Hofmeister series (Li^+^>Na^+^>K^+^>Cs^+^) when ordered by aggregate breaking strength. Here, at a given electrolyte concentration, the specific ion effects on aggregate stability can be explained by ion hydration effects, but amplification of the specific ion effects with decreasing electrolyte concentration cannot be explained by ion size or the ion hydration effect. It should be stressed that ion size and ion hydration effects often play important roles at high electrolyte concentration (>0.1mol L^−1^) [36,60]. In other words, the effects of the type of ion and its hydration radius can be ignored in low electrolyte concentration systems. In this way, specific ion effects should not occur at low electrolyte concentration. Obviously, the specific ion effects on aggregate stability shown in our experimental results (Figs 1 and 4) did not result from ion size and ion hydration effects. However, there are also at least two serious problems with these explanations. First, recent experimental and theoretical studies have confirmed that ions do not influence the structure of water beyond the first hydration shell, i.e., there is no long-range water ordering by ions [34,62,63]. Second, these explanations ignored the interface property and other hydrated solutes. For example, lysozyme precipitation follows reverse Hofmeister ordering when changing the pH or ionic strength of the bulk solution [62,64]. Therefore, although hydration effects help us understand some phenomena, this type of explanation is only coincidental and has been disproven [65].

Dispersion or quantum fluctuation forces, which are missing from classic theories, have recently been used to elucidate the mechanism of specific ion effects. Dispersion forces are quantum mechanical in nature and occur because of the interaction between the instantaneous dipoles of two ions or between the instantaneous dipoles of an ion and a surface. They depend on the nature of the ion, such as ion size and polarizability. Normally, with increasing ion size, ion polarizability increases and dispersion forces become stronger [23]. In this way, we can speculate that the dispersion forces of the alkali metal cations in the present work follow the order Cs^+^>K^+^>Na^+^>Li^+^. Strong ionic dispersion forces can induce ion adsorption to a surface, thereby screening the surface electric field [41]. Thus, based on dispersion force effects, we can infer that the aggregate stability follows the order Cs^+^>K^+^>Na^+^>Li^+^. Obviously, inference based on dispersion force effects can explain the occurrence of specific ion effects at a given electrolyte concentration in our work, but it cannot explain the amplification of specific ion effects with decreasing electrolyte concentration. Boström et al. [36] found that dispersion forces only become dominant at high electrolyte concentrations (>0.1 mol L^−1^). Therefore, with decreasing electrolyte concentration, specific ion effects would decrease, and at very low electrolyte concentrations, the specific ion effects may disappear if it is dispersion forces that produce specific ion effects. In contrast, our results showed that specific ion effects on aggregate stability sharply increased with decreasing electrolyte concentration, and at the lowest concentration of 10^−5^ mol L^−1^ the strongest specific ion effects were observed. In conclusion, ion size, hydration effects, or dispersion effects cannot properly explain the specific ion effects on soil aggregate stability.

In a previous paper, we found that the strong electric field from surface charges (usually >10^8^ V m^−1^) increased with decreasing electrolyte concentration, which determined the manner of soil aggregate swelling, dispersion, and explosion [16]. The surface electric field of clay particles increases with decreasing electrolyte concentration. It is interesting that the amplification of the specific ion effect with decreasing electrolyte concentration showed a similar relationship with the strong electric field. It is known that ions are polarized when they are in an electric field. For soil particles, the electric field at the particle surface is very strong (usually >10^8^ V m^−1^), and non-classic polarization of ions resulting from the coupling between the strong electric field and ionic quantum fluctuation may occur, which could produce strong non-classic induction between ions and the surface[25]. This seems to indicate that the origin of the specific ion effects on clay aggregate stability may originate from classic induction forces. However, it should be stressed that in classic theory the induction force is much weaker than dispersion forces. Because dispersion forces cannot explain the specific ion effects, the classic induction force alone cannot unravel the ion specificity. In the strong electric field of soils, ions will be highly polarized by coupling between quantum fluctuations of ionic extra-nuclear electrons and the electric field near surface, this could be referred to as non-classic polarization [46]. Thus, with the amplification of the coupling effects, the ability of ions to screen the surface electric field will be strong. In fact, Xu et al. [46] found that, the non-classic polarization would strongly influence clay aggregate stability. In the following section, we will employ the non-classic dipole moments of each cations in our experiments to quantitatively characterize the effects of cationic non-classic polarization on clay aggregate stability.

### Quantitative characterization of cationic non-classic polarization on clay aggregate stability

Based on the above experimental results and discussion, DLVO theory, which does not include ion size, hydration effects, or dispersion forces, cannot explain specific ion effects. Despite this, DLVO theory is still able to explain the experimental results in some simple scenarios. It can predict that with decreasing electrolyte concentration, the aggregate breaking strength increases for each individual ion species. For example, in the present study, for any given electrolyte system, the result that the aggregate breaking strength increases with decreasing electrolyte concentration in bulk solution can be explained by DLVO theory. Therefore, we suggest that classic DLVO theory is conditionally correct to explain the experimental results.

The question is under what conditions is DLVO theory appropriate. It is known that if the ion size effect, hydration effects, and dispersion forces are sufficiently weak that they can be reasonably ignored then DLVO theory can be applied. Therefore, in this study, we assumed that DLVO theory can be applied to the lithium cation. The reasons are as follows: (1) The radius of Li^+^ is the smallest among the four ion species and only 0.9 Å, and hence the ion size effect can be ignored. (2) Li^+^ has only two electrons in the first electron shell, which are strongly attracted to the positive atomic nucleus. In addition, its static polarizability is only 0.028 Å^3^, which is much lower than that of Na^+^ (0.139 Å^3^), K^+^ (0.814 Å^3^), and Cs^+^ (2.402 Å^3^), and therefore the ionic quantum fluctuation is quite weak [40]. (3) Ion size and ion hydration effects can be ignored at low electrolyte concentrations. Obviously, when Li^+^ approaches the clay surface, the coupling effects between the strong electric field and quantum fluctuations will be the weakest of the four cations considered in this study, and can thus be ignored. Therefore, it is reasonable to assume that the Li^+^ system can be properly explained by classic DLVO theory.

**Fig 5:**
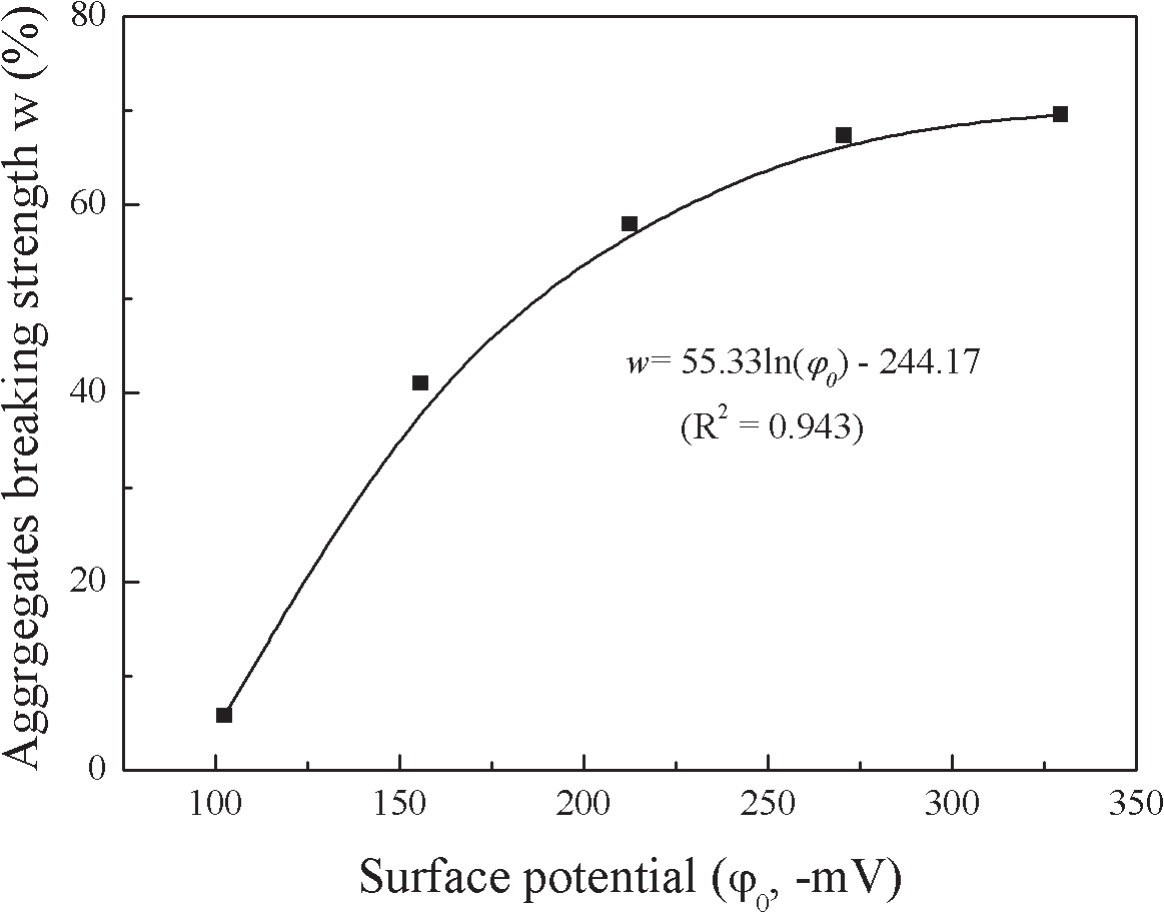
Relationship between the aggregate breaking strength (*d*<5 μm) and surface potential in Li^+^ system.

Because the Li^+^ system can be described by classic DLVO theory, we can establish the relationship between the aggregate breaking strength and the surface potential in the Li^+^ system (Fig 5). Here, we take the aggregate breaking strength of *d*<5 μm versus the surface potential as an example. The surface potential can be calculated with the following formula [66]:

$$\varphi_{0}=-\frac{2RT}{ZF}\ln\left(\frac{1-b}{1+b}\right)$$ 7

in which

$$\begin{array}{r} \frac{\kappa C_{T}}{Sc_{0}}=\;1+\frac{4}{1+b}-\frac{4}{1+e^{-1}b}\\ \kappa =\sqrt{\frac{8\pi F^{2}Z^{2}c_{0}}{\varepsilon RT}} \end{array}$$

where *φ*_0_ (V) is the surface potential of clay particles; *R* (J mol^−1^ K^−1^) is the universal gas constant; *T* (K) is the absolute temperature; *Z* is the valence of cation; *F* (C mol^−1^) is Faraday’s constant; *b* is the intermediate variable; ε is the dielectric constant, which is equal to 8.9×10^−10^ C^2^ J^−1^ dm^−1^ for water; *c*_0_ (mol L^−1^) is the equilibrium concentration of the cation in bulk solution; *S* (m^2^ g^−1^) is the specific surface area; and *C*_*T*_ (mol g^−1^) is the cation exchange capacity. The fitting results of aggregate breaking strength versus surface potential in the Li^+^ systems are shown in Fig 5.

According to DLVO theory, it is the electric field strength that determines the breaking strength. At a given surface potential for a given material, the electric field strength is constant for the Li^+^ or Na^+^, K^+^, and Cs^+^ systems. Therefore, for a given surface potential, the aggregate breaking strength would be the same for the Li^+^ or Na^+^, K^+^, and Cs^+^ systems. In other words, for the same electrolyte concentration of LiNO_3_, KNO_3_, NaNO_3_, and CsNO_3_, the difference in aggregate breaking strength reflects the difference in surface potential or electric field strength. These analyses showed that we could use the fitting equations shown in Fig 5 to estimate the surface potential of the clay particles in the Na^+^, K^+^, and Cs^+^ systems at each electrolyte concentration, and the results are shown in Table 1. From this table, even though the electrolyte concentration of each system is the same, the corresponding surface potentials are different. For instance, at the low electrolyte concentration of 10^−5^mol L^−1^, the absolute values of the surface potentials in the Li^+^, Na^+^, K^+^, and Cs^+^ systems were 329.4, 275.2, 128.4, and 95.8 mV, respectively. This shows that large differences in the surface potential occurred, and the surface potential in the 10^−5^ mol L^−1^ LiNO_3_ solution was 3.44 times higher than the potential in the 10^−5^ mol L^−1^ CsNO_3_ solution. However, according to classic double-layer theory, the surface potential in the 10^−5^ mol L^−1^ LiNO_3_ solution should be equal to the potential in the 10^−5^ mol L^−1^ CsNO_3_ solution. These results indicated that different monovalent cation species have different affinities for the clay surface and screen the electric field at the particle surface at different amounts, leading to different values of the surface potential. Cs^+^ has the highest affinity for the clay surface and screens the electric field at the particle surface the most, whereas Li^+^ has the lowest affinity and screens the electric field the least. The discussion in the above section indicated that these differences cannot be explained by the differences in dispersion forces, ion size, and hydration effects. The only possible explanation for the observed specific ion effects on clay aggregate stability is the strong non-classic polarization in the cation–surface interactions. In a strong electric field of >10^8^ V m^−1^, the adsorbed ion could be highly polarized [25,42]. Thus, the surface potentials of the clay particles shown in Table 1 for Na^+^, K^+^, and Cs^+^ are the surface potential when these cation species are polarized; and the stronger the polarization the lower the absolute value of the surface potential.

**Table 1:** Surface potentials of clay particles in Li^+^, Na^+^, K^+^ and Cs^+^ systems.

*C* (mol L^-1^)		*φ*_*0(i)*_ (-mV, *d*<5μm)		
	Li^+^	Na^+^	K^+^	Cs^+^
0.00001	329	257	128	95.8
0.0001	271	235	124	95.4
0.001	212	198	114	93.8
0.01	156	149	99.1	93.0
0.1	103	86.7	86.6	84.6

If the monovalent cation was polarized in the strong electric field near the montmorillonite surface, an additional Coulomb force from the dipole moment of the cation would be present. In other words, cation polarization will increase the Coulomb adsorption force between the cation and the surface. Thus, this effect could be taken as the change of the cation charge from *Z* to *βZ* (*β*≥ 1) [42]. *βZ* can be referred to as the effective charge number of a polarized cation, and *β* is the effective charge coefficient [42]. Therefore, *β* can be taken as a parameter to indirectly characterize the polarization intensity of a cation species. Obviously, because of polarization effects, the thickness of the diffuse layer, the surface potential, and the electric field strength near the surface would decrease.

If the surface potential based on classic theory (non-polarization) is *φ*_0(classic)_ and the surface potential based on polarization effects is *φ*_0(polar)_, then (*βZ*)*Fφ*_0(polar)_ = *ZFφ*_0(classic)_, or *β* = *φ*_0(classic)_/*φ*_0(polar)_. Here, *β* can be referred to as the absolute effective charge coefficient. In our study, we assumed that the polarizability of Li^+^ can be ignored and classic double-layer theory can be applied. Therefore, *φ*_0(classic)_ = *φ*_0(Li)_. However, when the polarization of Li^+^ is ignored, *β*_Li_ = 1, and thus *β*_*i/*Li_ = *β*_*i*_ */β*_Li_ = *β*_*i*_.

According to the surface potentials shown in Table 1, the *β* values can be calculated for each cation species, and the results are shown in Table 2. The *β* values obtained from the clay aggregate stability agree with the results of the *β* values from the cation exchange experiments. Liu et al. [42] obtained the relative *β* values of *β*_Na/Li_ = *β*_Na_/*β*_Li_ = 1.11 and *β*_K/Li_ = *β*_K_/*β*_Li_ = *β*_K_/*β*_Na_×*β*_Na_/*β*_Li_ = 1.646×1.11 = 1.827 as the electrolyte concentration approaches10^−3^–10^−2^ mol L^−1^. From the data shown in Table 2, the relative *β* values obtained from the aggregation stability were *β*_Na/Li_ = 1.074 and *β*_K/Li_ = 1.863 or *β*_Na/Li_ = 1.049 and *β*_K/Li_ = 1.574 at electrolyte concentrations of 10^−3^mol L^−1^ or 10^−2^mol L^−1^, respectively. The data in Table 2 shows that when the electrolyte concentration is in the range 10^−1^ of 10^−5^ mol L^−1^, with decreasing electrolyte concentration, the *β* values increase. Therefore, cation polarization increased with increasing electric field strength.

**Table 2:** The β value of different system under different electrolyte concentration (mol L^-1^).

β value	0.00001	0.0001	0.001	0.01	0.1
*β*_Na_	1.283	1.152	1.074	1.049	1.184
*β*_K_	2.569	2.192	1.863	1.574	1.186
*β*_Cs_	3.446	2.842	2.268	1.676	1.214

According to the *β* values in the range of the electrolyte concentration 10^−1^ to 10^−5^ mol L^−1^, we can calculate the average dipole moments of the *i*th adsorbed cation species in the diffuse layer using the following equation [25,42]:

$${\bar{p}}_{i}=\frac{\left(Z_{i}\beta_{i}-Z_{i}\right)F\varphi_{0}}{N_{0}\bar{E}}\approx \frac{\left(\beta_{i}-1\right)e}{\kappa }$$ 8

where ${\bar{p}}_{i}$ (D) is average dipole moment of the *i*th adsorbed cation species in the diffuse layer, *N*_0_ is Avogadro’s constant, and *e* is the electronic charge (1.6× 10^−19^ C). $\bar{E}$(V m^−1^) is the mean electric field strength in the diffuse layer:

E¯=κ∫01κE(x)dx=−κ[φ0−φ(1κ)] 9

*κ* is the Debye–Hückel parameter:

$$\kappa =\sqrt{\frac{8\pi {\left(Z_{i}^{}\beta_{i}\right)}^{2}F^{2}c_{\text{i}}^{0}}{\varepsilon RT}}=\beta_{i}\kappa_{\text{classic}}$$ 10

Where *κ*_classic_ is the Debye–Hückel parameter as the cation polarization is absent.

From the above equation, the cation polarization will greatly decrease the thickness (*l* = 1/*κ*) of the diffuse layer with *l* = *l*_classic_/*β*_*i*_. This indicates that the electric field near the particle surface could be strongly screened by cation polarization, leading to a decrease in the surface potential, as shown in Table 1.

The average dipole moment of the *i*th cation species (${{\bar{p}}^{\prime }}_{i}$) in the diffuse layer can be calculated using the intrinsic polarizabilities [25]:

$${{\bar{p}}^{\prime }}_{i}=4\pi \varepsilon_{0}\varepsilon_{w}\alpha_{i}^{}\bar{E}$$ 11

Where *ε*_0_ is the dielectric constant in a vacuum (8.85 × 10^−12^ C^2^ J^−1^ m^−1^), and *ε*_w_ is the static relative permittivity of water (81). *α*_*i*_ (Å^3^) is approximately equal to the intrinsic polarizability of the *i*th ion species, and *α*_Na_, *α*_K_, and *α*_Cs_ are equal to 0.139, 0.814, and 2.402 Å^3^, respectively [41].

It should be noted that all these numerical predictions obtained in the present study neglects the structure of the solvent molecules. The dielectric constant of water for bulk solution was used in all of the calculations. When the distance away from clay surface is less than 2 nm, the structure of the solvent molecules will be strongly changed and thus the dielectric constant of water will decrease. It can lead to strong hydration of clay surface, and the range of hydration force is only 1.5–2 nm. When the distance away from clay surface is more than 2 nm, however, the change of water structure could be ignored. It means the calculation of electrostatic field in this study is acceptable because the strong electrostatic force is crucial for soil aggregate breakdown when the distance is >2 nm [16].

Comparison of the average dipole moments between our experimental observations and classic calculations are shown in Table 3. The data in Table 3 shows that with increasing electronic shell number of the cation species, the average dipole moment increases. The order of electronic shell number is Cs^+^>K^+^>Na^+^, and the order of the average dipole moment is also Cs^+^>K^+^>Na^+^ for a given electrolyte concentration. This can be explained by the larger the electronic shell number, the weaker the binding force between the nucleus and the outer shell electrons, and thus the higher polarizability of the cation.

**Table 3:** The average dipole moment of *i*th cation species in diffuse layer.

Cation	0.00001 (mol L^-1^)		0.0001 (mol L^-1^)		0.001 (mol L^-1^)		0.01 (mol L^-1^)		0.1 (mol L^-1^)	
	${\bar{p}}_{i}\left(D\right)$	${{\bar{p}}^{\prime }}_{i}(D)$	${\bar{p}}_{i}\left(D\right)$	${{\bar{p}}^{\prime }}_{i}(D)$	${\bar{p}}_{i}\left(D\right)$	${{\bar{p}}^{\prime }}_{i}(D)$	${\bar{p}}_{i}\left(D\right)$	${{\bar{p}}^{\prime }}_{i}(D)$	${\bar{p}}_{i}\left(D\right)$	${{\bar{p}}^{\prime }}_{i}(D)$
Na^+^	1028	0.001272	194.4	0.003313	32.11	0.008195	6.884	0.01907	7.242	0.06030
K^+^	2846	0.007448	801.4	0.01940	215.9	0.04799	53.74	0.1117	7.308	0.3532
Cs^+^	3307	0.02198	955.1	0.05725	260.5	0.1416	59.44	0.3295	8.215	1.042

Here we would like to emphasize that, the DLVO theory neglects the ionic correlations which become important for di- or poly-valent counter-ions. In our study, just mono-valent cations were involved, thus that ignoring is possibly valid. Actually, a comparison between the ionic correlations with DFT and ionic non-classic polarization has shown that, the effect of ionic non-classic polarization is much stronger than the effect of ionic correlations [67].

It is clear that the adsorbed cations were strongly polarized in the strong electric field near the clay particle surface, and the strong polarization cannot be explained by classic polarization theory. Therefore, we refer to this as non-classic polarization. Obviously, the specific ion effect on clay aggregate stability resulted from non-classic polarization. If a cation species was strongly polarized, this strong polarization would increase the adsorption force between the cation species and the surface, increasing the screening effects of cations on the electric field near the clay surface. As a result, the electrostatic repulsive force between two adjacent particles in the aggregate decreased, and the stability of the aggregates therefore increased.

## Conclusions

Strong specific ion effects on clay aggregate stability were observed. This study showed that it was not ion size, hydration, or dispersion forces in the cation–surface interactions but strong non-classic polarization of the adsorbed cations that resulted in the specific ion effects. The strong polarization of the cations was caused by the strong external electric field arising from clay surface charges. We referred to this polarization as strong non-classic polarization because the observed dipole moments of the adsorbed cations could be up to 10,000 times larger than the classically calculated values for the same cation species. For example, with an electrolyte concentration of 10^−5^ mol L^−1^, the classically calculated average dipole moment of Cs^+^ in the diffuse layer was only 0.02198 D, but the observed dipole moment in our experiment was 3307 D. Furthermore, because the electric field near the particle surface increased with decreasing electrolyte concentration, the observed dipole moments sharply increased with decreasing electrolyte concentration. For example, when the electrolyte concentration decreased from 10^−1^ to 10^−5^ mol L^−1^, the average dipole moment of Cs^+^ increased from 8.215 to 3307 D. In addition, we found that with increasing electronic shell number of the cation species, the dipole moments increased, and increasing the electronic shell number inevitably increased the polarizability. Our study also indicated that, because of the strong polarization, the thickness of the diffuse layer significantly decreased, which enhanced the screening effects of adsorbed counter-ions on the electric field near the clay surface, which caused the surface potential to sharply decrease. For example, in 10^−5^ mol L^−1^ LiNO_3_ solution, the surface potential was −329 mV; however, in 10^−5^ mol L^−1^ CsNO_3_ solution, the surface potential sharply decreased to −95.8 mV.

We conclude that the strong polarization of the cation would increase the adsorption forces between the cation species and the clay surface, which would increase the screening effects of cations on the electric field near the clay surface. As a result, the electrostatic repulsive force between two adjacent particles in the aggregate decreases, and the stability of the aggregate therefore increases. Even though we only demonstrated specific ion effects on aggregate stability with several alkali ions, our results indicate that these effects would be universally important in soil aggregate stability.
